# Automated Landmark Detection and Lip Thickness Classification Using a Convolutional Neural Network in Lateral Cephalometric Radiographs

**DOI:** 10.3390/diagnostics15121468

**Published:** 2025-06-09

**Authors:** Miaomiao Han, Zhengqun Huo, Jiangyan Ren, Haiting Zhu, Huang Li, Jialing Li, Li Mei

**Affiliations:** 1 Department of Orthodontics, Nanjing Stomatological Hospital, Affiliated Hospital of Medical School, Research Institute of Stomatology, Nanjing University, Nanjing 210008, China; hmm10200201@163.com (M.H.); 18205082515@163.com (J.R.); 2 School of Internet of Things, Nanjing University of Posts and Telecommunications, Nanjing 210003, China; zqhuo123@163.com (Z.H.); htzhu@njupt.edu.cn (H.Z.); 3 Discipline of Orthodontics, Department of Oral Science, Faculty of Dentistry, University of Otago, Dunedin 9016, New Zealand; li.mei@otago.ac.nz

**Keywords:** deep learning, lip thickness, automated landmark localization, artificial intelligence

## Abstract

**Objective:** The objective of this study is to develop a convolutional neural network (CNN) for the automatic detection of soft and hard tissue landmarks and the classification of lip thickness on lateral cephalometric radiographs. **Methods:** A dataset of 1019 pre-orthodontic lateral cephalograms from patients with diverse malocclusions was utilized. A CNN-based model was trained to automatically detect 22 cephalometric landmarks. Upper and lower lip thicknesses were measured using some of these landmarks, and a pre-trained decision tree model was employed to classify lip thickness into the thin, normal, and thick categories. **Results:** The mean radial error (MRE) for detecting 22 landmarks was 0.97 ± 0.52 mm. Successful detection rates (SDRs) at threshold distances of 1.00, 1.50, 2.00, 2.50, 3.00, and 4.00 mm were 72.26%, 89.59%, 95.41%, 97.66%, 98.98%, and 99.47%, respectively. For nine soft tissue landmarks, the MRE was 1.08 ± 0.87 mm. Lip thickness classification accuracy was 0.91 ± 0.04 (upper lip) and 0.90 ± 0.04 (lower lip) in females and 0.92 ± 0.03 (upper lip) and 0.88 ± 0.05 (lower lip) in males. The area under the curve (AUC) values for lip thickness were ≥0.97 for all gender–lip combinations. **Conclusions:** The CNN-based landmark detection model demonstrated high precision, enabling reliable automatic classification of lip thickness using cephalometric radiographs.

## 1. Introduction

As a critical anatomical structure in the lower third of the face, the morphological characteristics of the lips play a decisive role in overall facial harmony. In facial esthetic evaluation, the proportional relationship between lip thickness and other facial structures is an essential quantitative indicator for assessing facial esthetics [1]. Changes in the upper lip following tooth movement have been widely studied. However, the extent of this change varies significantly depending on factors such as gender, age, ethnicity, soft tissue thickness, and lip strain [2]. Orthodontic treatment alters tooth position, which can in turn affect lip shape, thickness, length, and strain. The upper lip length and thickness have been found to increase after incisor retraction, with smaller increases observed in patients with thicker lips than those with thinner lips [3]. Thus, measuring lip thickness is essential for developing a customized orthodontic treatment plan for patient-centered healthcare.

In the literature, the thickness of the upper lip base is conventionally defined as the distance from the subnasal point to the subspinale point [4]. However, notable inconsistencies exist in the measurement methods for upper lip vermilion thickness [5,6]. Consequently, no standardized measurement protocol has been established for assessing the thickness of the upper lip, lower lip, or chin [7].

In orthodontics, cephalometric analysis plays a crucial role in clinical diagnosis, treatment planning, and outcome evaluation [8]. Lip thickness can be measured through soft and hard tissue landmark identification on lateral cephalometric radiographs [4,5]. However, landmark identification, measurement, and lip thickness calculation are time-consuming and labor-intensive for clinicians.

Artificial intelligence (AI), a core field in computer science, aims to develop systems and algorithms simulating human intelligence [8]. Deep learning technologies based on artificial neural networks exhibit powerful feature extraction and learning capabilities, with wide applications in image recognition, natural language processing, and decision optimization. As a core deep learning model, convolutional neural networks (CNNs) are designed to process grid-structured data (e.g., images), efficiently extracting spatial features via local perception, weight sharing, and pooling operations [9]. Currently, AI has been successfully applied in various dental domains, including caries detection, periapical lesion diagnosis, alveolar bone resorption assessment, cyst/tumor classification, cephalometric analysis, and osteoporosis screening [9,10,11,12]. In modern orthodontics, semi-automatic computer-assisted cephalometric measurement is widely used, where clinicians manually identify landmarks before software-based measurements [13]. However, this method remains highly dependent on clinicians’ expertise; thus, recent research has focused on developing fully automated AI-based cephalometric systems to enhance clinical efficiency and reduce subjective bias. Existing studies primarily target automated hard tissue landmark localization, but soft tissue landmark identification accuracy remains suboptimal due to challenges like low imaging clarity/contrast and anatomical variability [12].

Therefore, the first aim of this study was to automatically identify landmarks in lateral cephalometric radiographs, with a particular focus on the precise localization of soft tissue landmarks. The second aim was to measure and summarize the distribution characteristics of lip thickness in the East China population and establish a classification framework for lip thickness. The third objective was to achieve automatic lip thickness classification using a decision tree model based on the automatically identified landmarks, thus providing an efficient and objective assessment method for clinical applications.

## 2. Materials and Methods

**Figure 1:**
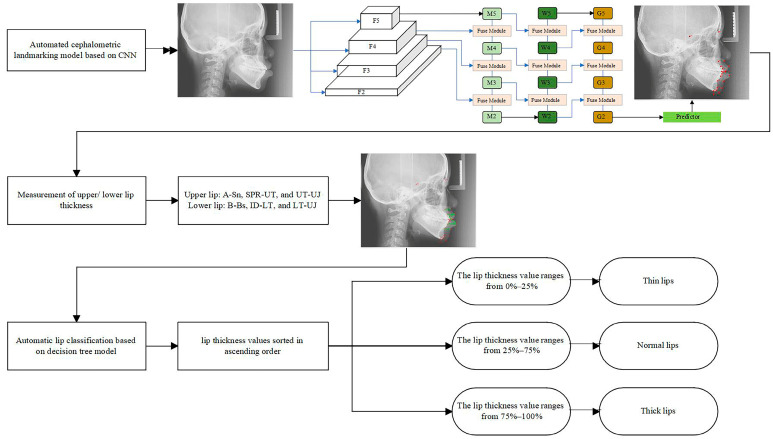
The experimental design of the present study. The red dots represent the auto-located output markers, and the green line segments indicate the lip thickness measurement lines.

This study was approved by the ethics committee of Nanjing Stomatological Hospital, Medical School of Nanjing University (approval number: NJSH-2024NL-063). The experimental design flowchart is shown in Figure 1.

### 2.1. Data Collection and Preprocessing

Data were collected from the radiology department of Nanjing Stomatological Hospital, Nanjing University, China. A total of 1019 subjects (318 males and 701 females) who sought orthodontic treatment at Nanjing University Stomatological Hospital between 2021 and 2024 were included in this study. All included subjects had lateral cephalograms as part of their medical records. Informed consent was obtained from all subjects. Lateral cephalometric radiographs were taken using a BDS221 Digital Panoramic Cephalometric System (Tuusula, Finland) from subjects with various malocclusions.

Inclusion criteria: (1) Patients with permanent dentition and Class I, II, or III malocclusions (regardless of severity) and (2) aged 18–45 years old.

Exclusion criteria: (1) History of craniofacial deformities, orthodontic treatment, orthognathic surgery, or facial plastic surgery (e.g., hyaluronic acid lip augmentation). (2) Cephalometric images obtained in non-intercuspal occlusion.

Imaging parameters included a tube voltage of 77 kV, a tube current of 16 mA, an exposure time of 8 s, and a distance of 140 cm from the center of the X-ray tube to the sensor. Subjects were instructed to (1) maintain a natural head position, (2) keep their head stable, (3) relax and breathe calmly, and (4) avoid chewing or swallowing during image acquisition. All images were saved in .jpg format.

Annotation personnel: A single orthodontist with 15 years of clinical experience annotated 200 lateral cephalometric films to establish a gold standard and standardized annotation criteria for two additional orthodontists. The two orthodontists subsequently identified landmarks on the remaining 819 lateral cephalometric images. The two orthodontists recorded the X and Y coordinates of each landmark on all radiographs. Intraclass correlation coefficients (ICCs) were calculated separately for each landmark’s X and Y coordinates.

The formula is as follows:

$$\mathrm{ICC}=\frac{Interdoctor\;variance}{Interdoctor\;variance+error\;variance}$$

**Figure 2:**
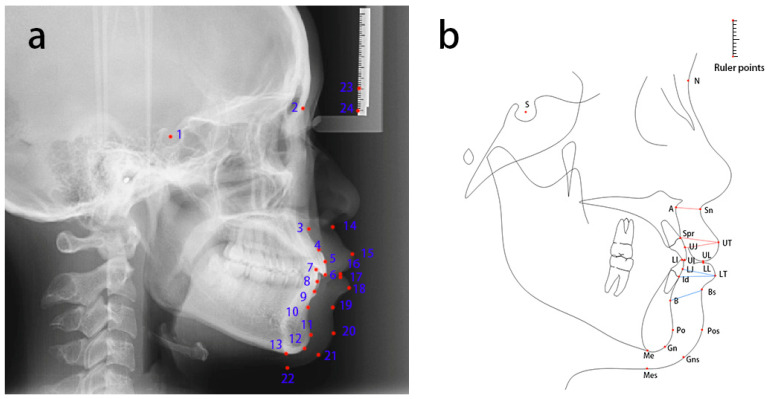
Location instructions and definition of the 24 cephalometric landmarks on the cephalogram (**a**) and schematic (**b**). The measurement segments for the upper lip are A-Sn, Spr-UT, and UT-UJ, as shown in red in (**b**). For the lower lip, they are B-Bs, Id-LT, and LT-LJ, as shown in blue in (**b**).

Annotation: A total of 24 landmarks on lateral cephalometric radiographs were identified using the Labelme (version 5.0.1) software. These include nine soft tissue landmarks: Sn, UT, UL, LT, LL, Bs, Pos, Gns, and Mes; thirteen bone tissue landmarks: S, N, A, Spr, UJ, UI, LI, LJ, Id, B, Po, Gn, and Me (Figure 2a, Table 1); and two ruler points. Upper lip thickness was measured as the average length of three line segments: A-Sn, Spr-UT, and UT-UJ [4,5,6]. Due to the lack of consensus in the literature on lower lip thickness measurement methods, the current study defined lower lip thickness as the average length of three line segments: B-Bs, Id-LT, and LT-LJ (Figure 2b).

Image Processing: The collected lateral cephalometric radiographs were cropped to a uniform size of 800 pixels by 640 pixels, and personal patient information (e.g., ID number and name) was removed. Each pixel corresponded to 0.2 mm. During the training phase of the automatic cephalometric landmarking model, data augmentation operations, such as random contrast enhancement, random histogram equalization, and random half-body flipping, were employed.

### 2.2. The Automatic Cephalometric Landmark Detection Model

**Figure 3:**
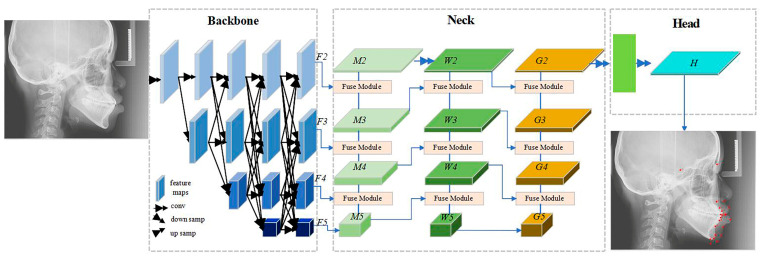
The network architecture of the automatic cephalometric landmark detection model. The framework follows the backbone–neck–head structure. The red dots indicate the auto-located output markers.

The network architecture of the automatic cephalometric landmark detection model follows the backbone–neck–head structure, as shown in Figure 3. HRNet [14] is selected as the backbone network to extract multi-scale features. After inputting the lateral cephalogram image, the backbone network extracts multi-scale feature maps, which will be fused in the neck later. The neck is designed based on SRPose [15], and the feature fusion module uses separable convolutional layers to fuse features. The neck module adopts a structure similar to the feature pyramid network (FPN) [16] to fuse multi-scale features, including two feature fusion paths from small-scale to large-scale feature maps (M5 to M2 and G5 to G2) and one feature fusion path from large-scale to small-scale feature maps (W2 to W5). The head utilizes SRPose and super-resolution technology to encode and upsample the fused features into high-resolution heatmaps. After obtaining the high-resolution heatmaps, the coordinates of the landmark points are determined by finding the peak positions in the heatmaps.

The Adam optimizer was used during model training with an initial learning rate of 0.0001. The learning rate was reduced by a factor of ten every 50 epochs. The batch size was set to 2, and training was conducted for 300 epochs. Various data augmentation techniques were applied to mitigate overfitting due to limited training data, including random contrast enhancement, histogram equalization, and half-body flipping. Each training sample underwent all augmentation steps in sequence, with randomized parameters for each operation (e.g., rotation angle and contrast intensity). These augmentations enhanced the diversity of the training data, improving model generalization.

### 2.3. Model Evaluation and Accuracy Assessment

After the automatic cephalometric landmark detection model is trained, the test datasets are input into the model, and the predicted heatmaps for each key point are obtained. The coordinates of landmarks are recovered by performing non-maximum suppression (NMS) over the predicted heatmaps [17]. The evaluation metrics used include the mean radial error (MRE, in millimeters; the smaller, the better) and the successful detection rates (SDR; the larger, the better) within radii of 1.00, 1.50, 2.00, 2.50, 3.00, and 4.00 mm. MRE is the average distance between predicted and ground-truth landmarks, and SDR is the percentage of predicted landmarks within a pre-defined range of ground-truth landmarks.

The formulas are as follows:

$$R=\sqrt{∆x_{i}^{2}+∆y_{i}^{2}}$$

$$\mathrm{MRE}=\sum_{i=1}^{n}R_{i}$$

$$\mathrm{SD}=\sqrt{\frac{\sum_{i=1}^{n}{\left(R_{i}-MRE\right)}^{2}}{N-1}}$$

$$\mathrm{SDR}=\frac{N_{acc}}{N_{all}}$$

where ${∆x}_{i}$ and ${∆y}_{i}$ represent the absolute differences between the predicted and ground-truth coordinates of the landmark on the X-axis and Y-axis, respectively, and *n* is the total number of landmarks. $N_{acc}$ and $N_{all}$ represent the number of correctly predicted landmarks and the total number of landmarks within the radius ranges (1.00, 1.50, 2.00, 2.50, 3.00, and 4.00 mm), respectively.

### 2.4. The Decision Tree Model

The classification method for lip thickness was based on previous studies [18]. The upper and lower lip thickness values of 1019 patients were measured and then sorted in ascending order for each gender. The first 25% were classified as the thin-lip group, the middle 50% (25–75%) as the normal-lip group, and the last 25% as the thick-lip group. The overall performance of the decision tree model was evaluated using receiver operating characteristic (ROC) curves, the area under the curve (AUC), accuracy, sensitivity, specificity, precision, and the F1 score. The metrics above were calculated using the true positive (TP), false positive (FP), true negative (TN), and false negative (FN).

The calculation formula is as follows:

$$\mathrm{Accuracy}=\frac{TP+TN}{TP+TN+FP+FN}$$

$$\mathrm{Recall}=\mathrm{Sensitivity}=\frac{TP}{TP+FN}$$

$$\mathrm{Specificity}=\frac{TN}{TN+FP}$$

$$\mathrm{Precision}=\frac{TP}{TP+FP}$$

$$F1\;\mathrm{score}=\frac{2\times Precision\times Recall}{Precision+Recal}$$

## 3. Results

### 3.1. Accuracy of Automatic Landmark Localization

**Figure 4:**
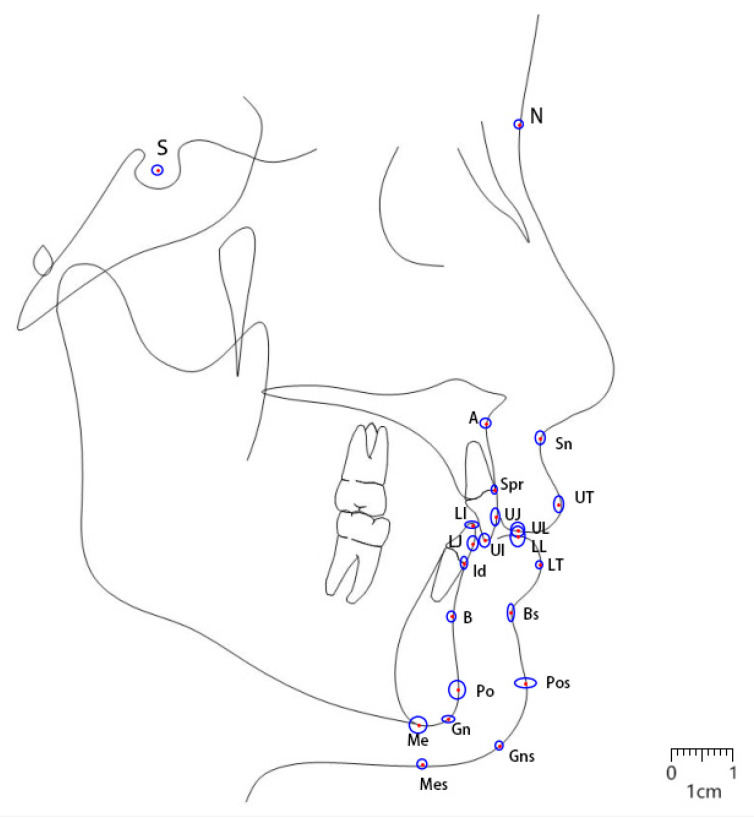
Error distribution of each AI-predicted landmark along the X-axis and Y-axis (using manually annotated landmarks as the coordinate origin). Red indicates manually located markers, and blue indicates AI-located markers. The coordinate distribution of AI-predicted landmarks is shown, along with an ellipse representing the 95% confidence interval.

**Figure A1:**
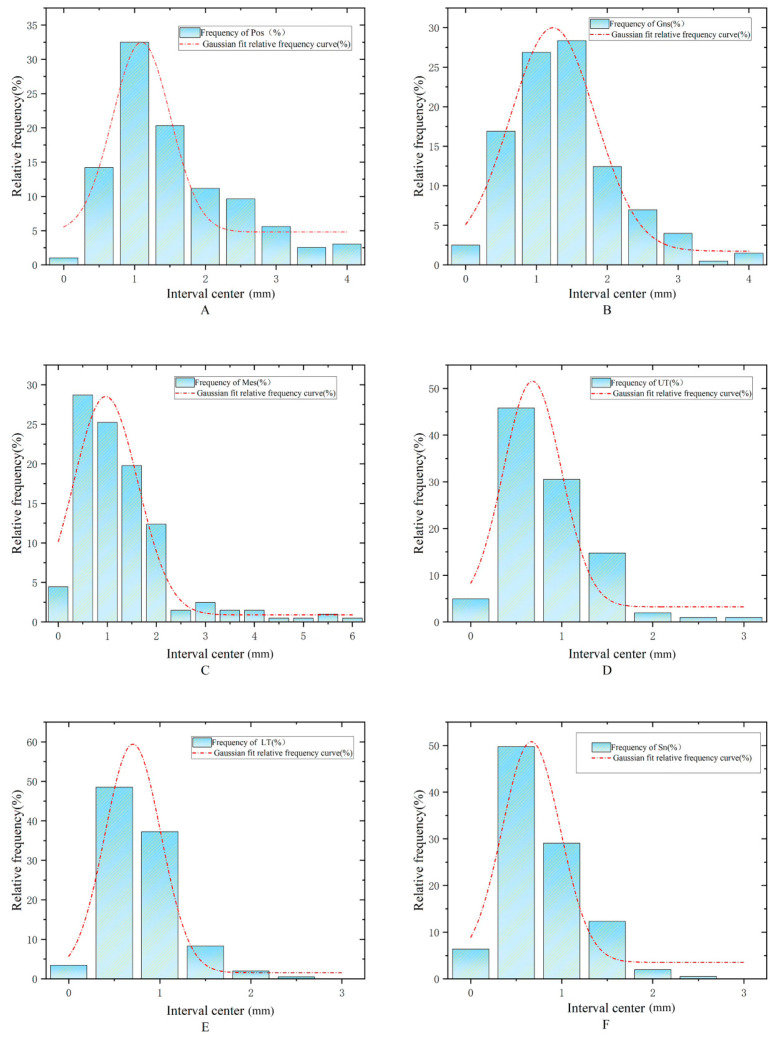
(**A**–**F**) sequentially depict the cumulative frequency distribution and Gaussian fitting curves for the soft tissue landmarks: Pos, Gns, Mes, UT, LT, and Sn.

**Figure A2:**
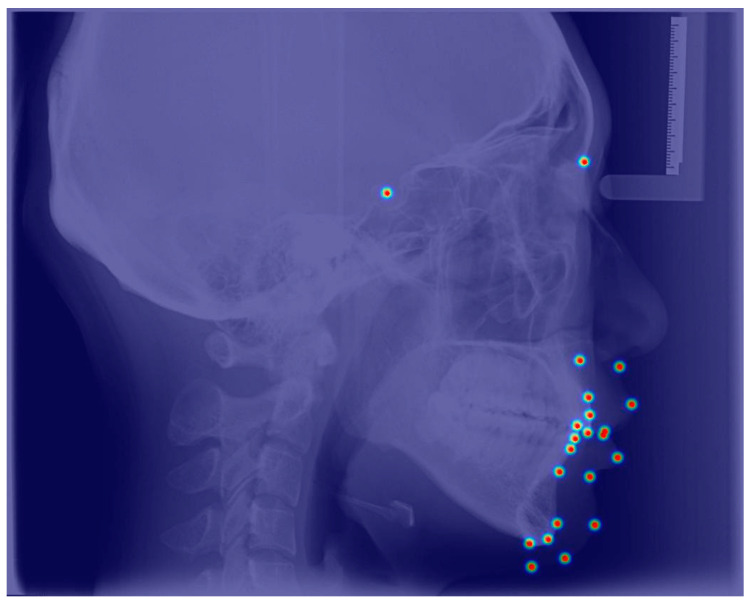
Visualized landmark heatmaps output by the automated landmark detection model.

The two orthodontists’ intraclass correlation coefficient (ICC) for landmark locations exceeded 90%. The automatic cephalometric landmark detection model demonstrated a rapid automatic landmarking speed, taking an average of 0.13 s to recognize and locate 22 landmarks in each lateral cephalometric X-ray image. Excluding ruler point 1 and ruler point 2 (which were not included in the training), the remaining 22 landmarks were successfully identified in each image, resulting in a recognition success rate of 100%. The mean radial error (MRE) for the 22 cephalometric landmarks was 0.97 ± 0.52 mm. The successful detection rates (SDRs) within various error margins were as follows: 72.26% within 1.00 mm, 89.59% within 1.50 mm, 95.41% within 2.00 mm, 97.66% within 2.50 mm, 98.98% within 3.00 mm, and 99.47% within 4.00 mm (Table 2). Using the manually annotated landmark coordinates as the reference, we obtained the coordinate distribution of AI-predicted landmarks and plotted an ellipse representing the 95% confidence interval, as shown in Figure 4. Additionally, we calculated the discrepancies between the machine-annotated and manually annotated landmarks and analyzed the relative frequency distribution of these discrepancies. The Gaussian-fitted curve provided a more precise visualization of the error concentration and distribution characteristics (Appendix A Figure A1). Visualized landmark heatmaps output by the automated landmark detection model is shown in Appendix A Figure A2.

**Figure A3:**
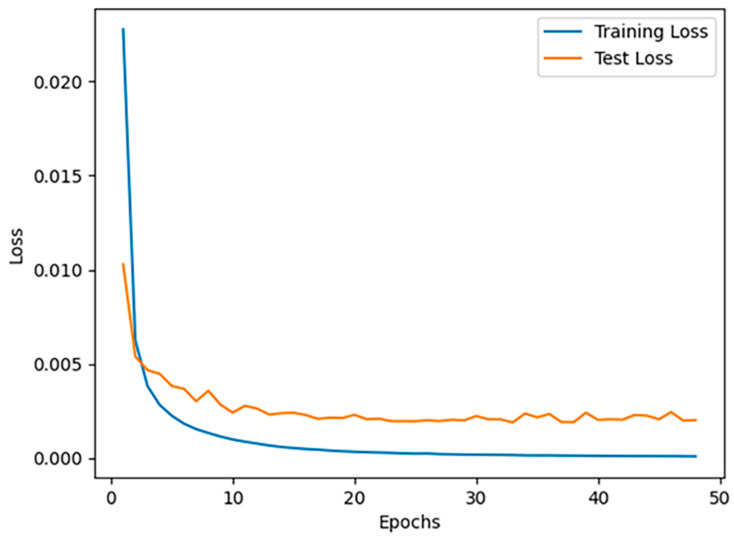
The loss curves of landmark detection models in test datasets and training datasets.

The loss curves of the landmark detection model are shown in Appendix A Figure A3. During the first 10 epochs, both the training and test losses decreased rapidly. By the 50th epoch, the losses approached zero and stabilized, indicating that the performance of the landmark detection model progressively improved, approaching human-level precision automatically.

### 3.2. Lip Thickness Classification Performance

**Figure 5:**
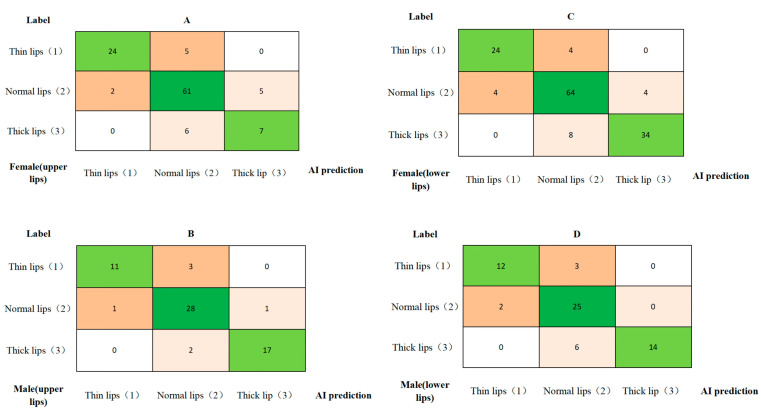
The confusion matrices for upper and lower lip thickness classification in females and males: (**A**) upper lip thickness in females, (**B**) upper lip thickness in males, (**C**) lower lip thickness in females, and (**D**) lower lip thickness in males. The x-axis represents the AI-predicted lip thickness classification, while the y-axis represents the manually annotated lip thickness classification. The green color indicates agreement between manual and AI-based lip thickness classifications; other colors indicate disagreement.

The classification standards for the lip thickness of the upper and lower lips based on different genders were established (Table 3). The measured lip thickness values were categorized according to the preliminary lip thickness classification criteria. Confusion matrices for the three categories, which comprise both the AI-predicted lip thickness classification and the manually annotated lip thickness classification, are shown in Figure 5.

**Figure 6:**
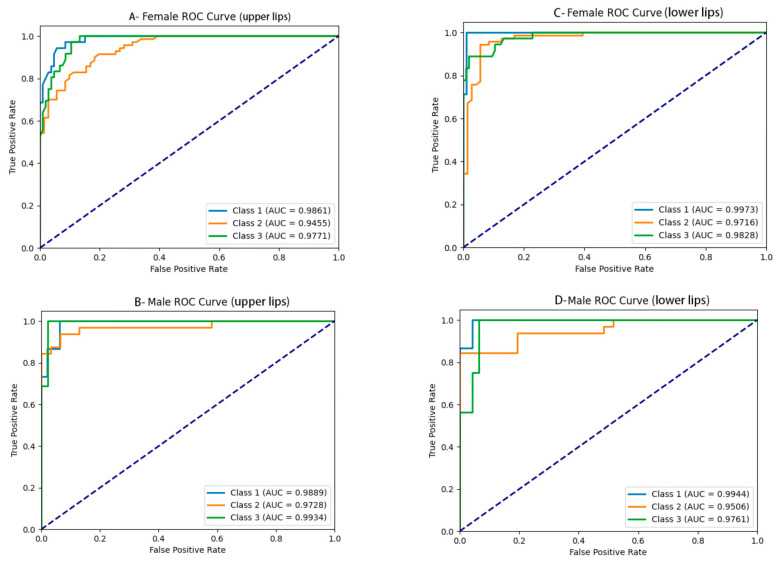
The ROC curves for upper and lower lip thickness classification in females and males: (**A**) upper lip thickness in females, (**B**) upper lip thickness in males, (**C**) lower lip thickness in females, and (**D**) lower lip thickness in males.

The performance of the upper and lower lip thickness classification for males and females is as follows: excellent accuracy (0.88–0.93), sensitivity and recall (0.81–0.87), specificity (0.90–0.94), precision (0.86–0.90), and F1 score (0.82–0.88) (Table 4). The AUC values were calculated for each class, and the ROC curves were generated, as shown in Table 4 and Figure 6. The AUC values were as follows: For upper lip thickness in females and males, they were (0.97 ± 0.02) and (0.98 ± 0.01), respectively. For lower lip thickness in females and males, they were (0.98 ± 0.01) and (0.98 ± 0.02), respectively.

## 4. Discussion

This study focuses on automatic landmark detection in pre-treatment patients’ lateral cephalograms and the classification of lip thickness. It provides a data-driven foundation for personalized orthodontic treatment planning. By assessing lip thickness before treatment, the model can predict how much a patient’s lateral profile may change following extraction orthodontic treatment [18]. For example, in patients with thick lips, the lateral profile is less impacted by retraction. As a result, greater anterior teeth retraction can be performed to enhance facial harmony. In contrast, patients with thin lips are more likely to experience significant changes in their lateral profile, so minimal retraction is recommended to avoid lip area collapse and premature facial aging [19]. This predictive capability helps clinicians select treatment strategies that balance functional correction with esthetic outcomes.

Multiple studies have explored the application of deep learning using cephalometric analysis. For example, Kang et al. reported a mean radial error (MRE) of 1.45 ± 0.92 mm for 18 anatomical landmarks [20], while Yao et al. noted the highest localization error at the Pos landmark (2.03 ± 5.95 mm) [21]. Hong et al. used a deep learning network based on reinforcement learning and found that the successful detection rate of landmarks within 2 mm was 67.33% [22]. Using an automatic program, Bao et al.’s study [23] showed that the mean radial error (MRE) for 19 landmarks was 2.07 ± 1.35 mm. The average success detection rates (SDRs) within 1 mm, 2 mm, 2.5 mm, 3 mm, and 4 mm were 18.82%, 58.58%, 71.70%, 82.04%, and 91.39%, respectively. By comparison, our model achieved an MRE of 1.08 ± 0.87 mm specifically for nine soft tissue landmarks, indicating improved precision, particularly in regions typically associated with higher localization error. This comparison highlights the performance advantage of our model and supports its effectiveness in enhancing soft tissue landmark detection in lateral cephalometric radiographs. Furthermore, based on the lip thickness data of 1019 patients with various types of malocclusion, this study preliminarily summarized the characteristics of lip thickness in the East China population and proposed a lip thickness classification framework. Additionally, the thicknesses of the upper and lower lips were measured, and automated lip thickness classification was achieved using a decision tree model based on the automatically detected landmarks.

Various factors can influence the changes in the soft tissue of the lips. For example, tooth extraction and non-extraction approaches can affect lip thickness at the lip base and vermilion; extraction treatment has been shown to cause more significant increases [24]^.^ Different ethnicities may also respond differently to treatment, influenced by lip tension and dental crowding [24]. Previous studies have mainly focused on the ratio of incisor retraction to soft tissue changes [25,26,27,28,29], neglecting individual differences in lip thickness, a crucial factor influencing facial protrusion and esthetic outcomes [30]. Facial soft tissue thickness has been found to differ between sexes, with males generally having thicker soft tissue than females [29]. When predicting soft tissue changes, factors such as lip thickness, lip strain, mandibular divergence, incisor position, gender, and ethnicity should be considered.

Over the past few decades, a large number of computer vision and machine learning techniques have been applied to automated medical image landmark detection. In the early stage, researchers defined landmarks as corner points, endpoints, local extreme points, etc., and designed image filters, operators, and template-matching algorithms to address this problem [31]. However, these models generally exhibited poor generalization performance. With the development of deep-learning technology, CNN-based methods have demonstrated superior performance in landmark detection tasks. The mainstream approaches can be classified into direct landmark location regression and landmark heatmap estimation [32].

Direct landmark location regression uses CNN to extract image features and then directly maps these image features to landmark coordinates via the fully connected network (FCN). However, soft tissue on lateral cephalometric radiographs is usually complex, making it challenging to map CNN-extracted features directly to landmark coordinates. In contrast, landmark heatmap estimation is more effective for soft tissue landmark detection [33]. This approach also employs CNN for feature extraction. The key difference lies in that it maps features to heatmaps and finally determines landmark coordinates by identifying the highest heat values within these heatmaps.

Among soft tissue landmarks, the Pos point had the most significant positioning error. It represents the most anterior point of the soft tissue in the chin. This point is easily located in a normally developed chin due to its prominence. However, the soft tissue in the chin slopes without apparent protrusion in patients with skeletal Class II malocclusion and a vertical growth pattern, making Pos point localization difficult. The highest error was observed at the soft tissue of the menton, possibly because of the thin chin soft tissue and indistinct edge delineation. The detection challenges for these soft tissue landmarks include low contrast in soft tissue radiographs, limited imaging clarity, and high anatomical variability. To address these issues, it is essential to develop a deep learning model that possesses enhanced resistance to interference and adaptability across different scenarios. This model should be capable of effectively handling anomalies and challenging samples, ultimately improving overall performance.

In this study, for hard tissue landmark localization, the mean radial error (MRE) of point B was higher than that of other hard tissue landmarks. Anatomically, the B point represents the deepest concavity of the mandible; it is easily identifiable in individuals with normal chin development due to the distinct curvature, but it is uncertain in cases of retrognathia or insufficient chin growth. The error at point A is attributed to overlapping anatomical structures, obscuring the deepest point of the maxilla in imaging. The nasopalatine suture (N point) may not always appear in radiographic images. Overlapping bilateral structures likely cause the error in Po point localization. This finding aligns with previous studies [34] indicating that landmarks such as A, ANS, B, Po, and Pos are relatively difficult to identify.

There are also limitations in our study. Although this study included 1019 patients with various malocclusions, the relatively limited geographic distribution of the sample may restrict the generalizability of the findings to the overall lip thickness characteristics of the Chinese population. Future research should explore multi-center validation to enhance the robustness of the findings. For example, the proposed model could automatically validate cephalometric point localization on lateral cephalograms from diverse populations across multiple hospital settings. Conducting such multi-center studies would substantially improve the reliability, generalizability, and scientific impact of the results.

As the first systematic investigation to summarize and explore lip thickness distribution in the East China population, this study represents a significant contribution with innovative value. In the future, the proposed system can be integrated either as a standalone tool or within widely used clinical cephalometric analysis software, e.g., Dolphin. Automatic landmark detection can be performed on radiographs from diverse patient populations across multiple clinical sites, followed by manual verification and correction by trained clinicians. To ensure accuracy and improve model generalizability, the system is designed to log manual adjustments, which can be used to retrain and refine the model iteratively. We also outline preliminary user training protocols and propose routine quality control procedures, including inter- and intra-operator consistency checks, to support safe and effective clinical deployment.

## 5. Conclusions

The automatic cephalometric landmark detection model with a backbone–neck–head network structure demonstrated high accuracy in detecting cephalometric landmarks, especially for soft tissue landmarks. By using the automatically detected landmarks, the thicknesses of the upper and lower lips can be measured. When these measurements are input into the decision tree classification model, automatic lip classification can be accomplished. This novel automated landmark detection and lip thickness classification method holds great potential for application in orthodontic clinical practice.

## Figures and Tables

**Table 1:** Cephalometric landmarks used in this study.

No.	Landmarks
1	Sella (S)
2	Nasion (N)
3	Subspinale (A)
4	Superior prosthion (Spr)
5	The most labial surface of the upper incisor (UJ)
6	Upper incisor (UI)
7	Lower incisor (LI)
8	The most labial surface of the lower incisor (LJ)
9	Infradentale (Id)
10	Supramental (B)
11	Pogonion (Po)
12	Gnathion (Gn)
13	Menton (Me)
14	Subnasale (Sn)
15	Labrale superius (UT)
16	Stomion superius (UL)
17	Stomion superius (LL)
18	Labrale inferius (LT)
19	Inferior labial sulcus (Bs)
20	Pogonion of soft tissue (Pos)
21	Gnathion of soft tissue (Gns)
22	Menton of soft tissue (Mes)
23	Ruler point 1
24	Ruler point 2

**Table 2:** The success detection rates and MRE for 22 landmarks ($\bar{x}$ ± s).

Landmark		Success Detection Rates (%)						Mean ± SD (mm)
		1 mm	1.5 mm	2 mm	2.5 mm	3 mm	4 mm	
***Soft tissue***								
	Sn	75.98	95.59	99.02	100.00	100.00	100.00	0.71 ± 0.41
	UL	75.49	92.65	98.04	98.53	99.51	100.00	0.76 ± 0.46
	UT	76.96	95.10	96.57	97.55	99.02	99.51	0.85 ± 0.47
	LL	79.41	93.63	98.04	99.51	100.00	100.00	0.70 ± 0.45
	LT	83.82	95.10	99.51	99.51	100.00	100.00	0.73 ± 0.37
	Bs	78.92	92.65	96.57	100.00	100.00	100.00	0.79 ± 0.49
	Pos	34.31	61.27	78.92	85.78	91.18	96.57	1.54 ± 1.09
	Gns	38.73	66.18	83.82	91.67	95.59	98.53	1.34 ± 0.83
	Mes	50.00	75.00	86.76	89.71	92.16	95.59	2.50 ± 1.27
***Hard tissue***								
	S	84.31	97.55	99.51	100.00	100.00	100.00	0.66 ± 0.37
	N	72.55	90.69	96.08	97.55	99.02	99.02	1.95 ± 1.56
	A	59.31	84.31	95.59	98.04	98.53	100.00	0.96 ± 0.56
	Spr	87.75	98.04	99.51	100.00	100.00	100.00	0.64 ± 0.32
	UJ	86.76	99.02	100.00	100.00	100.00	100.00	0.65 ± 0.30
	UI	86.27	96.57	99.02	99.51	100.00	100.00	0.65 ± 0.38
	LI	79.41	94.12	96.57	99.02	99.02	99.51	0.78 ± 0.52
	LJ	84.31	95.59	98.53	99.02	99.51	99.51	0.72 ± 0.43
	Id	85.78	96.08	98.53	100.00	100.00	100.00	0.69 ± 0.35
	B	61.27	83.82	90.69	96.08	98.04	100.00	1.02 ± 0.78
	Po	60.29	81.86	93.14	99.51	100.00	100.00	0.98 ± 0.55
	Gn	75.49	95.59	98.04	99.51	100.00	100.00	0.77 ± 0.41
	Me	72.55	90.69	96.57	98.04	99.51	100.00	0.83 ± 0.52
**Average**		72.26	89.59	95.41	97.66	98.69	99.47	0.97 ± 0.52

**Table 3:** Upper and lower lip thicknesses in male and female subjects in different lip thickness classifications (mm).

Classification	Upper Lip		Lower Lip	
	Female	Male	Female	Male
Thin lip	6.69–10.14	6.84–10.81	7.72–11.51	8.88–11.47
Normal lip	10.15–12.64	10.83–13.93	11.52–13.74	11.49–14.61
Thick lip	12.67–15.98	13.96–20.88	13.75–17.07	14.67–20.35

**Table 4:** The performance of the classification for upper and lower lip thicknesses.

		Accuracy	Sensitivity	Specificity	Precision	F1-Score	AUC
**Upper lip thickness**							
Female		0.91 ± 0.04	0.86 ± 0.04	0.92 ± 0.07	0.88 ± 0.04	0.87 ± 0.01	0.97 ± 0.02
	Class 1	0.95	0.82	0.98	0.92	0.87	0.98
	Class 2	0.87	0.89	0.84	0.84	0.87	0.94
	Class 3	0.92	0.86	0.94	0.88	0.87	0.97
Male		0.92 ± 0.03	0.87 ± 0.76	0.93 ± 0.07	0.90 ± 0.05	0.88 ± 0.04	0.98 ± 0.02
	Class 1	0.93	0.78	0.97	0.91	0.84	0.98
	Class 2	0.88	0.93	0.84	0.84	0.88	0.97
	Class 3	0.95	0.89	0.97	0.94	0.91	0.99
**Lower lip thickness**							
Female		0.90 ± 0.04	0.85 ± 0.02	0.91 ± 0.07	0.86 ± 0.03	0.85 ± 0.01	0.98 ± 0.01
	Class 1	0.94	0.85	0.96	0.85	0.85	0.99
	Class 2	0.85	0.88	0.82	0.84	0.86	0.97
	Class 3	0.91	0.80	0.96	0.89	0.85	0.98
Male		0.88 ± 0.05	0.80 ± 0.11	0.90 ± 0.14	0.86 ± 0.13	0.82 ± 0.04	0.98 ± 0.02
	Class 1	0.91	0.80	0.95	0.85	0.82	0.99
	Class 2	0.82	0.92	0.74	0.73	0.81	0.95
	Class 3	0.90	0.70	1.00	1.00	0.82	0.97

## Data Availability

The datasets supporting the conclusions of this article are presented within the article. The raw data can be obtained from the corresponding author upon reasonable request.
